# Psychiatric consultations in cardiology units: an eight-year retrospective analysis

**DOI:** 10.1192/j.eurpsy.2026.12019

**Published:** 2026-07-31

**Authors:** S. Florek, A. Suchodolski, M. Szulik, R. Pudlo

**Affiliations:** 1 Department of Psychoprophylaxis, Faculty of Medical Sciences in Zabrze, Medical University of Silesia, Tarnowskie Góry; 2 Department of Cardiology and Electrotherapy, Silesian Centre for Heart Diseases, Zabrze; 3 Department of Psychoprophylaxis, Faculty of Medical Sciences in Zabrze, Medical University of Silesia, Tarnowskie Góry, Poland

## Abstract

**Introduction:**

Mental disorders are frequent among patients with cardiovascular diseases and negatively influence prognosis. Despite the clinical relevance, data on the characteristics of psychiatric consultations in cardiology wards are scarce.

**Objectives:**

To describe the prevalence, profile and outcomes of psychiatric consultations carried out in cardiology departments of a tertiary reference centre over an eight-year period.

**Methods:**

A retrospective review of psychiatric consultations performed between January 2016 and March 2024 at the Silesian Centre for Heart Diseases, Zabrze, was conducted. From 2,196 records, 1,447 met inclusion criteria (adult inpatients, complete data, consultation performed in cardiology, intensive cardiac care, mechanical circulatory support or rehabilitation units). Demographic data, cardiac ICD-10 diagnoses, previous and newly initiated psychopharmacotherapy, and consultations addressing legal/ethical issues or the need for early re-evaluation were analysed using descriptive statistics and Spearman correlations.

**Results:**

The sample comprised 491 women (34%) and 956 men (66%), mean age 60.9 ± 16.6 years. Most consultations occurred in cardiology wards (n = 782). Heart failure (I50) was the most frequent cardiac diagnosis (n = 525), followed by acute myocardial infarction (I21). New psychopharmacological treatment was recommended in 34.9% of cases, most often in heart failure and arrhythmia patients. Women predominated among subjects with aortic valve disease (I35), atrioventricular block (I44) and atrial fibrillation/flutter (I48), many of whom were already receiving psychiatric therapy. Consultations regarding capacity to consent or ethical/legal issues, and recommendations for psychiatric reassessment within four weeks, were more common in older patients and in those with heart failure. Correlations between age and most clinical variables were weak (Spearman ρ ≤ 0.3).

**Conclusions:**

Psychiatric morbidity is highly prevalent among hospitalised cardiology patients. One third of consultations resulted in initiation of psychopharmacotherapy, emphasising the importance of routine psychiatric involvement. Heart failure and arrhythmias accounted for most referrals, while capacity assessments and early follow-up were mainly required in elderly individuals. Collaboration between cardiologists and psychiatrists is essential to optimise outcomes in this complex population.

**Disclosure of Interest:**

None Declared

